# Validation of the modified Fresno Test: assessing physical therapists' evidence based practice knowledge and skills

**DOI:** 10.1186/1472-6920-10-38

**Published:** 2010-05-25

**Authors:** Julie K Tilson

**Affiliations:** 1 Division of Biokinesiology and Physical Therapy, University of Southern California, Los Angeles, California, USA

## Abstract

**Background:**

Health care educators need valid and reliable tools to assess evidence based practice (EBP) knowledge and skills. Such instruments have yet to be developed for use among physical therapists. The Fresno Test (FT) has been validated only among general practitioners and occupational therapists and does not assess integration of research evidence with patient perspectives and clinical expertise. The purpose of this study was to develop and validate a modified FT to assess EBP knowledge and skills relevant to physical therapist (PT) practice.

**Methods:**

The FT was modified to include PT-specific content and two new questions to assess integration of patient perspectives and clinical expertise with research evidence. An expert panel reviewed the test for content validity. A cross-sectional cohort representing three training levels (EBP-novice students, EBP-trained students, EBP-expert faculty) completed the test. Two blinded raters, not involved in test development, independently scored each test. Construct validity was assessed through analysis of variance for linear trends among known groups. Inter and intra-rater reliability, internal consistency, item discrimination index, item total correlation, and difficulty were analyzed.

**Results:**

Among 108 participants (31 EBP-novice students, 50 EBP-trained students, and 27 EBP-expert faculty), there was a statistically significant (p < 0.0001) difference in total score corresponding to training level. Total score reliability and psychometric properties of items modified for discipline-specific content were excellent [inter-rater (ICC (2,1)] = 0.91); intra-rater (ICC (2,1)] = 0.95, 0.96)]. Cronbach's α was 0.78. Of the two new items, only one had strong psychometric properties.

**Conclusions:**

The 13-item modified FT presented here is a valid, reliable assessment of physical therapists' EBP knowledge and skills. One new item assesses integration of patient perspective as part of the EBP model. Educators and researchers may use the 13-item modified FT to evaluate PT EBP curricula and physical therapists' EBP knowledge and skills.

## Background

Evidence based practice (EBP) is the integration of the best available research evidence with clinical expertise and patients' unique perspectives and circumstances to optimize healthcare outcomes[1]. EBP knowledge and skills have become foundational principles for all health care professionals[2]. The introduction in 1992 of formal methods for teaching evidence based medicine[3] prompted health care educators to integrate EBP core principles into their curricula[4-8]. To guide and measure this transformation, educators need comprehensive, valid, and practical instruments to assess learners' EBP knowledge and skills.

Educators are encouraged to develop EBP curricula that address the 5-step model described in the Sicily Statement on Evidence Based Practice[2] as core principles of EBP (Table 1). A comprehensive EBP knowledge and skills assessment should be based on this 5-step model. Although over one hundred instruments for evaluating EBP curriculum effectiveness have been identified[9], only one - the Fresno Test (FT)[10] - has established validity and reliability and covers a broad range of EBP knowledge and skills.

**Table 1:** The 5-step EBP model[2]

Step	Activity
**Step 1**	**Ask: ** Translation of uncertainty into a focused, searchable clinical question

**Step 2**	**Acquire: ** Search for and retrieval of research evidence

**Step 3**	**Appraise: ** Critical appraisal of research evidence for validity and clinical importance

**Step 4**	**Apply: ** Integration of research evidence with patient perspectives and clinical expertise; application of appraised evidence to practice

**Step 5**	**Assess: ** Evaluation of performance/reflection

The original FT consists of two clinical scenarios, 7 short answer questions, and 5 fill-in-the-blank questions that assess knowledge and skills from steps 1-3 of the EBP model. Scoring the FT is based on a rubric with descriptions and examples of "excellent", "strong", "limited", "minimal" and "not evident" answers for each question. The instrument and scoring rubric are discipline-specific, and the psychometric properties of the original FT have been reported only for family medicine residents and faculty members[10].

The FT is a commonly used outcome measure of EBP knowledge and skills[11-13]. However, because it is discipline-specific, use in disciplines other than family medicine require modification and validation. The 7-item *adapted *FT[14] developed for occupational therapists demonstrated acceptable psychometric properties and was responsive to change in EBP-novice occupational therapist learners. The adapted FT includes occupational therapy-specific clinical scenarios and scoring rubric examples. In addition to modifying discipline-specific content, the instrument developers deleted 5 fill-in-the-blank questions because the educational intervention under investigation did not address the topics assessed by those items (statistical calculation skills and knowledge about diagnostic and prognostic study design)[14]. However, many EBP curricula include these topics[15-19] and the 5 deleted items demonstrated strong psychometric properties in the original FT[10]. Hence, the consequence of deleting the 5 fill-in-the-blank items is to narrow the instruments' assessment of core EBP principles for many curricula.

The physical therapy profession has embraced the inclusion of EBP in professional curricula[20,21]. To date however, only self-report instruments have been developed to assess EBP knowledge and skills among physical therapists[22,23]. Neither the original FT (specific to family medicine physicians), nor the adapted FT (specific to occupational therapists), is appropriate for assessment of physical therapists. Assessment of EBP curricula effectiveness in physical therapy education requires the development of a valid and reliable assessment of physical therapists' EBP knowledge and skills.

The original and adapted versions of the FT assess only steps 1-3 of the EBP model (ask, acquire, appraise); they do not assess step 4--the ability to integrate patient perspectives and clinical expertise with the best available research evidence[24]. Knowledge and skills for integrating patient perspectives and clinical expertise with research evidence are integral to the definition and central premise of EBP[1]. Failing to assess this knowledge sends an implicit message to learners that it is not important. Although other aspects of EBP (*e.g.*, self-reflection [step 5], behaviour, beliefs, and care outcomes) are best assessed by other instruments, it is reasonable to expect the FT to address the core principles of EBP knowledge and skills from 4 of the 5 steps of the EBP model.

The purpose of this study was to develop and validate a modified FT to assess physical therapists' EBP knowledge and skills. Discipline-specific content of the original FT was modified and two questions were added to more comprehensively assess core EBP principles described in the 5-step model.

## Methods

### Modified Fresno Test Development

Development of the modified FT consisted of three phases: 1) discipline-specific modification, 2) development of new items, and 3) establishment of content validity.

#### Phase 1: Discipline-specific modification

The author identified all elements (scenarios, questions, grading rubric) of the original FT requiring discipline-specific modification. The essential components of each element requiring modification were identified and recorded and a template was developed for discipline-specific modification. For example, the fundamental structure for Clinical Scenario 1 was identified as:

3-4 sentences that:

◦ *Introduce a discipline-specific patient, problem, and brief salient history (e.g. chronic vs. acute)*

◦ *Provide information that clarifies the patient's diagnosis*

◦ *Introduce the primary objective for treatment*

◦ *Introduce a potential intervention and comparison that the practitioner wants to know more about*

Physical therapy-specific content was developed following this template. Discipline-specific content requiring modification consisted of 3 clinical scenarios, 4 question stems, and the scoring rubric for 5 items.

#### Phase 2: Instrument expansion

To expand the scope of the original FT, the author developed two short answer questions and a corresponding scoring rubric (Additional File 1). Item 8 was designed to assess knowledge and skills associated with acquiring information about patient perspectives and circumstances. Item 9 was designed to assess knowledge and skills associated with integration of clinical expertise into evidence-based clinical decision making. These items were then modified in response to expert panel feedback (see Phase 3).

#### Phase 3: Content validity

Content validity of the modified FT was established through formal feedback from four PT EBP experts. The panelists were PT EBP educators representing diverse academic and geographic settings (panelist 1: entry-level doctoral education, Western US; panelist 2: entry-level masters education, Eastern Canada; panelist 3: post-professional education, Northeastern US; panelist 4: clinical educator, Southeastern US). Panelist feedback addressed item clarity, difficulty, and importance.

The modified FT's content validity was supported by consensus among the expert panel that the test is a comprehensive assessment of important EBP knowledge and skills for physical therapists. However, the panel recommended several changes. First, the panel suggested that the wording of items 1-7 required clarification. The 7-item adapted FT[14] included modifications to items 1-7 that addressed the panel's concerns about clarity. Therefore, the wording and structure presented in the adapted FT was adopted for items 1-7 including reduction of the number of clinical scenarios (3 to 2) and the number of clinical questions (3 to 1). Second, two panelists believed that the calculation items were too difficult or not pertinent to physical therapy practice and research literature. To address this concern, the value of calculation items was reduced from 32 to 28 points and one calculation (Negative Predictive Value) was replaced with a question requiring interpretation of an alpha and p-value. The new version of the test contained 14 items (9 short answer, 5 fill-in-the-blank, and 232 possible points). Table 2 illustrates the categories of EBP knowledge and skills assessed. Additional File 1 contains the complete test and scoring rubric.

**Table 2:** Modified and Original Fresno Tests: Percentage and point allocation to EBP steps 1-4

EBP Step	Original Fresno Test	14-item Modified Fresno Test	13-item Modified Fresno Test*
**Step 1: Ask**	11% (24)	10% (24)	11% (24)

**Step 2: Acquire**	23% (48 )	21% (48)	21% (48)

**Step 3: Appraise ** **(Qualitative questions)**	49% (104 )	45% (104)	46% (104)

**Step 3: Appraise ** **(Quantitative questions)**	17% (36 )	14% (32)	14% (32)

**Step 4: Apply**	--	10% (24 )	7% (16 )

**Total Points**	212	232	224

Reported as percentage of total score (point value)

* Recommended version for physical therapists. Item 9 removed from the "Step 4: Apply" category secondary to unacceptable psychometric properties.

EBP: Evidence Based Practice

### Test takers

A cross-sectional convenience sample (n = 108) representing 3 EBP training levels (EBP-novice PT students, EBP-trained PT students, and EBP-expert PT faculty) completed the test. EBP-novice students were first year of Doctor of Physical Therapy (DPT) students (n = 96) at the University of Southern California (USC). EBP-trained students were third year DPT students (n = 91) at USC. EBP-expert faculty were recruited through an email list-serve for PT educators (general-list@aptaeducation.org; approximately 700 members). PT educator respondents affirmed EBP expertise and experience teaching EBP prior to completing the test. Table 3 details each group's EBP training and experience. This study was approved by the University of Southern California Institutional Review Board (HS-07-00465) and all participants gave informed consent to participate.

**Table 3:** Characteristics of 3 groups of test takers with known levels of EBP training

Group	n					Amount, topic, and timing of EBP training prior to testing
**EBP-novice ** **PT students**	**31**					**4 months prior:**
						▪ Introduction to EBP (2 hours)
						▪ Introduction to searching (2 hours) (PubMed)
						▪ Introduction to appraisal (2 hours)
						**1 week prior:**
						▪ How to write a searchable clinical question (1 hour)

**EBP-trained ** **PT students**	**50**					**3 years prior:**
						▪ Introduction to EBP (6 hours per above)
						▪ EBP course 1 (question development, searching, appraisal, application to practice; 32 hours)
						**2 years prior:**
						▪ EBP course 2 (introduction to statistics; 32 hours)
						**Previous year:**
						▪ Integration of EBP into advanced and clinical course work (time varied)

**EBP-expert ** **PT faculty**	**27**					**Years teaching EBP: Percentage of faculty participants**
						▪ 1-2 years: 7%
						▪ 3-5 years: 41%
						▪ 6-10 years: 31%
						▪ > 10 years: 21%

PT = physical therapist, EBP = evidence based practice

### Raters

Two individuals, experienced in teaching EBP to physical therapy students and not involved in test development, served as raters for the study. Rater training occurred in three parts. First, both raters attended a 2-hour author-lead introduction to the modified FT, the scoring rubric, and a standardized data collection form. During the meeting, the author reviewed and discussed scoring for a sample test. Next, each rater spent 2.5 hours rating 5 pilot tests including samples from each of the three EBP training groups. During this practice period, the author was available for consultation and questions. Finally, an additional 1.5 hour author-lead meeting was held during which raters and the author compared and discussed scores for the pilot tests. Score discrepancies were explored and resolved.

Both raters independently scored each test and re-scored 22 randomly selected tests two weeks later. Raters were masked to each other's scores and to their own scores for re-test reliability. The retest number of 22 was selected based upon a power analysis recommended by Walter *et al.*[25] for reliability studies given α = 0.05 and β = 0.20 for a null ICC = 0.50 and anticipated ICC = 0.80.

### Testing

The author used commercially available survey software (^©^1999-2009 SurveyMonkey.com) to administer the test and to download de-identified data to a database. Test takers were allowed up to 60 minutes to complete the test. No external resources were permitted except for a calculator and note paper. An open text field for voluntary participant comments was provided at the conclusion of the test. Student test-takers were supervised in a computer lab. Faculty completed the test remotely and confirmed an honor statement before and after the test stating that they did not use external resources.

### Data Analysis

Inter and intra-rater reliability were calculated using intraclass correlation coefficient [ICC (2,1)] for total score and individual item analysis. ICC values were interpreted as: excellent reliability ≥0.8, moderate reliability = 0.60-0.79, and questionable reliability < 0.60[26]. Internal consistency was calculated using Cronbach's α. Known groups validity was determined by analysis of variance for linear trends.

The author defined a passing score as > 50% of available points for individual items. The passing score was intentionally set lower than the passing score defined as "mastery of the material" by Ramos *et al.*[10], to reduce the risk of a floor effect among EBP-novice students. Item discrimination index (IDI) was calculated for each question by separating participants' total scores into quartiles and then subtracting the proportion of participants in the bottom quartile who passed that item from the proportion of participants in the top quartile who passed the same item[27]. IDI ranges from -1.0 to 1.0 and represents the difference in passing rate between test takers with high overall scores (top 25%) and low overall scores (bottom 25%). IDI > 0.2 was considered acceptable[27].

Correlation between item score and total score, corrected item-total correlation (ITC), was assessed using Pearson product-moment correlation coefficients. ITC > 0.3 was considered acceptable[28]. Item difficulty was characterized by calculating the proportion of test takers who achieved a passing score for each item. Chi-square analysis was used to compare individual item pass rates by group; a p-value < 0.05 was considered statistically significant. Data were analyzed using SPSS (Version 16.0).

## Results

A total of 108 individuals (31 EBP-novice PT students, 50 EBP-trained PT students, and 27 EBP-expert PT faculty) enrolled in the study and completed the modified FT.

### Total score reliability

Modified FT inter-rater reliability was excellent: ICC (95% Confidence Interval) = 0.91 (0.87 - 0.94). Intra-rater reliability was excellent for both raters: Rater 1 ICC = 0.95 (0.90 - 0.98); Rater 2 ICC = 0.96 (0.90 - 0.98). Internal consistency was acceptable (Cronbach's α = 0.78)[29].

### Known groups validity

**Figure 1:**
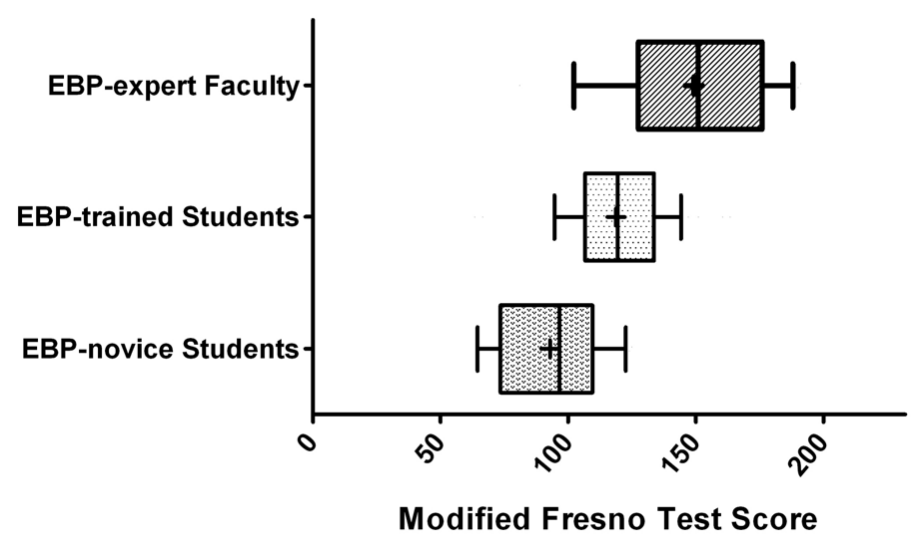
**Modified Fresno Test scores by group**. Box and whisker plot of modified Fresno Test scores for EBP-novice PT Students (n = 31), EBP-trained PT Students (n = 50), and EBP-expert PT Faculty (n = 27). The central box spans from the lower to the upper quartile, the middle line represents the median, the '+' sign represents the mean, the whiskers extend from the 10^th ^percentile to the 90^th ^percentile of scores. Analysis of variance for linear trends was p < 0.0001.

The three groups with known differences in EBP training had distinct differences in total score on the modified FT (Figure 1). There was a statistically significant linear trend (p < 0.0001) for sequentially improved mean score by group corresponding to level of training: EBP-novice students, 92.8 (40.0% total points); EBP-trained students, 118.5 (51.1%); EBP-expert faculty, 149.0 (64.2%). Absolute difference in performance between EBP-novice and EBP-trained students was 11.1% (25.7 points) and between EBP-trained students and EBP-expert faculty was 13.1% (30.5 points).

### Individual item analysis

Table 4 describes individual item results for inter-rater reliability, intra-rater reliability, IDI, ITC, and item difficulty (pass rate). Intra-rater reliability was moderate to excellent for both raters for all items (ICC = 0.62 - 1.0). Inter-rater reliability was moderate to excellent (ICC = 0.61 - 0.99) for all items with the exception of items 8 and 9 (the two new items) which had ICC = 0.47 and 0.41, respectively. IDI was acceptable (> 0.2) for all items. ITC was acceptable (> 0.3) for all items with the exception of item 9 (ITC = 0.20). There was a statistically significant difference between groups for item pass rate with the exception of items 1, 2, and 9.

**Table 4:** Psychometric properties by individual test item

	Reliability					Pass Rate				
				
Item	Inter-rater (n = 108)	Intra-rater 1 (n = 22)	Intra-rater 2 (n = 22)	IDI (n = 108)	ITC (n = 108)	All (n = 108)	Novice (n = 31)	Trained (n = 50)	Expert (n = 27)	p-value†
**1**	0.92	0.97	0.95	0.39	0.44	75%	68%	74%	85%	0.30

**2**	0.90	0.83	0.96	0.46	0.40	71%	68%	74%	70%	0.83

**3**	0.89	0.95	0.89	0.64	0.65	56%	32%	60%	78%	< 0.01

**4**	0.83	0.87	0.81	0.68	0.64	80%	52%	92%	89%	< 0.01

**5**	0.73	0.96	0.81	0.61	0.52	56%	39%	56%	74%	0.03

**6**	0.69	0.77	0.62	0.46	0.52	76%	61%	72%	100%	< 0.01

**7**	0.78	0.91	0.72	0.54	0.66	24%	3%	18%	59%	< 0.01

**8**	0.47	0.62	0.95	0.61	0.46	36%	42%	78%	81%	< 0.01

**9**	0.41	0.76	0.78	0.25	0.20	65%	58%	66%	70%	0.60

**10**	0.99	0.99	1.0	0.50	0.57	17%	3%	2%	59%	< 0.01

**11**	0.96	0.97	0.96	0.29	0.62	10%	0%	4%	33%	< 0.01

**12**	0.61	0.91	0.67	0.39	0.41	20%	10%	12%	48%	< 0.01

**13**	0.91	1.0	1.0	0.54	0.48	25%	19%	14%	52%	< 0.01

**14**	0.94	1.0	1.0	0.54	0.44	38%	16%	32%	74%	< 0.01

† Chi-square analysis for difference in pass rate between EBP-novice PT students, EBP-trained PT students, and EBP-expert PT faculty

IDI = Item Discrimination Index, ITC = Item-total correlation

### Post hoc analysis

*Post hoc *analysis of a 13-item modified FT (item 9 removed) demonstrated enhanced total score reliability and internal consistency (Table 5).

**Table 5:** Total score reliability for 14-item and 13-item versions of the modified FT

	14-item Modified FT	13-item Modified FT (item 9 removed)
Intra-rater Reliability*	0.91 (0.87 - 0.94)	0.92 (0.88 - 0.94)

Intra-rater Reliability* (Rater 1)	0.95 (0.90 - 0.98)	0.96 (0.91 - 0.98)

Intra-rater Reliability* (Rater 2)	0.96 (0.90 - 0.98)	0.96 (0.91 - 0.98)

Internal Consistency**	0.78	0.79

* Reported as ICC (95% Confidence Interval)

** Reported as Cronbach's alpha

### Time to completion and participant comments

All participants completed the test within the 60 minutes allotted. Minutes to test completion (mean ± standard deviation) were: EBP-novice students, 33.2 ± 8.7; EBP-trained students, 34.8 ± 10.0; and EBP-expert faculty, 40.5 ± 15.5. Twenty-one test-takers (19%) volunteered feedback about the test. Of those, 12 (5 EBP-trained students, 7 EBP-expert faculty) commented that access (e.g. internet access) to formulas for statistical calculations should be permitted.

## Discussion

This study demonstrates that the modified FT is a valid and reliable instrument for assessing EBP knowledge and skills among physical therapists. The modified FT provides a physical therapy-specific assessment of core principles identified in steps 1 through 4 of the EBP model. Previous versions of the FT[10,14] do not assess knowledge and skills for integrating patient perspective and clinical expertise in EBP. One new question, associated with integration of patient perspectives in EBP, demonstrated satisfactory psychometric properties and should be included in the modified FT. With consideration of the limitations discussed below, educators and researchers are encouraged to use the 13-item version of the modified FT to evaluate PT EBP curricula and physical therapists' EBP knowledge and skills.

### Total score reliability

Total score reliability was excellent for two independent, blinded raters, unfamiliar with any version the FT prior to this study. Previous versions of the FT have also demonstrated excellent reliability. However, raters for the original FT were involved in development of the test and scoring rubric[10] and the adapted FT required scoring rubric revision before acceptable reliability was achieved[14]. Hence, the modified FT demonstrated excellent reliability under more rigorous and generalizable conditions than has been previously reported.

Rater training may have contributed substantially to the test's reliability. Nine of the 14 items require subjective scoring based on a complex rubric (Additional File 1). Use of pilot tests provided an opportunity for clarification of scoring procedures and may be an essential ingredient for achieving inter-rater reliability. Additionally, scoring the modified FT is time intensive and reliability may be dependent on raters having sufficient time to complete the scoring process. Scoring time of 10-15 minutes per test should be allocated for practiced scorers.

### Validity

The considerable difference in performance for each successive EBP training group (EBP-novice PT students, EBP-trained PT students, and EBP-expert PT faculty) provides strong support for the construct validity of the modified FT. Scores for the EBP-novice and EBP-expert groups are comparable to novice and expert scores on the original FT[10]. Expanding upon previous work, the modified FT maintained discriminative validity under the more challenging condition of a third, mid-level training group (EBP-trained PT students).

It is important to consider that the differences between groups could have been influenced by clinical experience. However, no floor effect was observed among EBP-novice students who had some EBP training but no clinical experience. Likewise, the EBP-expert cohort, with extensive clinical experience, did not demonstrate a ceiling effect. This indicates that EBP knowledge and skills--not clinical experience--was the primary construct being tested.

Longitudinal studies are needed to understand the modified FT's responsiveness to change over time. McCluskey and Bishop[14] considered a 10% change on the adapted FT to be educationally important. The difference in mean scores between successive groups in this study exceed 10% suggesting that the test has potential for responding to change in EBP skills over time.

### Individual items: discipline-specific modification

Generally, the 12 items modified for discipline-specific content from the original FT (items 1-7 and 10-14) demonstrated strong psychometric properties (*i.e., *moderate to excellent inter and intra-rater reliability, acceptable IDI and ITC, statistically significant difference in pass rates between groups). The only deficits noted were the absence of a statistically significant difference in group pass rates for item 1 (ask a focused question) and item 2 (sources for evidence). These two items had acceptable IDI scores. This suggests that although the items discriminate between high and low performing test takers (IDI), they do not assess knowledge or skills that are distinctly different among groups with known differences in EBP training. This trend is evident in previous versions of the FT[10,14] and may indicate that developing a focused clinical question and evaluating sources of evidence are mastered early in EBP education.

The items requiring statistical calculations (items 10-12) were among the most difficult for all groups. This does not make the items of poor value. The psychometric properties for these items were acceptable and the items provide important insight into the effectiveness of quantitative components of curricula. To minimize arithmetic errors, future versions of the modified FT should use 'natural frequency'[30] values that test-takers can compute without need for a calculator. For example, item 10 of the current version requires calculation of sensitivity using the values '9' and '29' (sensitivity = 9/29 = 31%). Natural frequency values such as '10' and '30' (sensitivity = 10/30 = 33%) are easier to compute.

Test takers suggested that access outside resources, specifically to reference statistical equations for items 10-12, would enhance the test's ecological validity (as most clinicians have routine access to the internet). The author views this suggestion with caution. First, asking test takers to recall information (including equations) assesses a deeper level of knowledge than can be achieved with open access to resources. Second, allowing access to outside resources, namely the internet, would impact the validity of all test items. However, given the overall poor performance on items involving calculations, a compromise would be to provide the equations within the test while continuing to restrict general access to outside resources. This change would require additional validation.

### Individual items: new content

The new items (8 and 9) of the modified FT were developed to address incongruence between the definition of EBP[1] and the contents of the original FT. Standard curricula teach that EBP is the integration of best available research evidence, clinical expertise, and patient perspectives and circumstances. Although the original FT addresses learners' knowledge and skills for finding and appraising the best available research evidence, it does not address the integration of clinical expertise and patient perspectives and circumstances.

The new item that assessed learners' ability to obtain information about patient perspective (item 8) demonstrated strong psychometric properties with the exception of inter-rater reliability. Questionable inter-rater reliability was not unexpected given that previously developed FT items and their corresponding scoring rubrics have had the benefit of repeated testing and modification. Given the value added by the content assessed and the overall psychometric properties, inclusion of this item (8) for future use of the modified FT is recommended with enhanced attention to rater training to facilitate inter-rater reliability.

The new item that assessed learners' ability to integrate clinical expertise (item 9) demonstrated questionable inter-rater reliability, unacceptable IDI and ITC, and did not demonstrate a statistically significant difference in pass rate between groups. In its current form, item 9 cannot be recommended for future use. However, the intended topic of assessment remains important. Future testing of the item with the addition of a clinical scenario that establishes a clear need to integrate clinical expertise and research evidence is warranted.

### Limitations

This study has three primary limitations to consider. First, the expert panel was limited to four individuals who were only consulted at the beginning of test development. The four individuals represented diverse PT EBP educational environments, however, a larger panel would have added to the generalizability of the test's content validity. Additionally, a more iterative process wherein the test was sent back to the panel to gain additional consensus and feedback would have strengthened the study design.

Second, practicing clinicians with limited EBP training were not included in the sample population. Therefore, generalization to this important cohort of learners is limited. The 7-item adapted FT[14] was tested among practicing occupational therapists[13]. Given that the adapted FT was sensitive to change among therapists with low scores at baseline but not those with high scores, the more comprehensive modified FT may demonstrate enhanced sensitivity to change among intermediate and advanced clinician learners.

Third, as discussed previously, one of the new items demonstrated unsatisfactory psychometric properties. This item is not recommended for future use without modification and retesting. Fortunately, the item represents a small percentage of the overall modified FT score and the impact of removing it is only to strengthen already robust reliability and validity results. Future work is needed to effectively assess skills associated with the integration of clinical expertise as a component of the EBP model.

## Conclusions

The modified FT is a valid, reliable assessment of core principle EBP knowledge and skills for physical therapists. The 13-item modified FT expands on the original FT by addressing integration of patient perspective as part of the EBP model. Scoring the modified FT is time intensive; recommendations for rater training are provided. Educators and researchers are encouraged to use the 13-item version of the modified FT to assess PT EBP curricula and physical therapists' EBP knowledge and skills.

## Competing interests

The author declare that they have no competing interests.

## Authors' contributions

JT conducted all phases of the study: design, data collection, data analysis, writing, and approval of the manuscript.

## Pre-publication history

The pre-publication history for this paper can be accessed here:

http://www.biomedcentral.com/1472-6920/10/38/prepub

## Supplementary Material

Additional file 1 Modified Fresno Test for physical therapists with scoring rubric. Click here for file
