# Supplementary material for: Validation of the modified Fresno Test: assessing physical therapists' evidence based practice knowledge and skills
Source: BMC Med Educ. 2010 May 25;10:38. doi: 10.1186/1472-6920-10-38 (PMC2896364; doi:10.1186/1472-6920-10-38)
Supplement: Additional file 1 — Modified Fresno Test for physical therapists with scoring rubric. [file 1472-6920-10-38-S1.DOC]

**Additional File 1**

**Part A. 13-item modified Fresno Test and scoring rubric**

**Instructions:**
Evidence Based Practice (EBP) involves knowledge and skills related to identifying and evaluating evidence to inform practice. This tool, the modified Fresno Test is designed to assess your EBP skills.

There are 8 short answer questions, 2 questions that require a series of mathematical calculations, and 3 fill-in-the-blank questions. A calculator and note paper have been provided for you. Additional resources (internet sites, books, etc) are not permitted.

Please complete the entire test in one sitting and allow yourself up to 60 minutes to complete the test.

-------------------------------------------------------------------------------------------------------------------------------------------------------

Answer questions 1-4 and 8 based on the following clinical scenarios:

**Scenario 1:** You have just evaluated Mary, a secretary who recently experienced a work related low-back injury moving 10, 25 lbs. file boxes 3 days ago. Her radiographs are negative and her only symptom is resolving 2/10 pain across the low back with forward bending and prolonged sitting. She has been off of work for 2 days and is eager to return but is also anxious about re-injury. You are considering a stabilization exercise program but wonder if manual therapy should be included in the patient’s physical therapy program.

**Scenario #2:** Marvin is a 10 year-old boy with hemiparesis secondary to stroke associated with an Arterial-Venous Malformation. He presents to outpatient therapy and his parents express particular concern about Marvin’s arm and leg weakness. You are considering implementing an intensive task-specific strengthening program but a colleague warns that such a program is likely to increase the patient’s moderate flexor tone and spasticity and suggests low intensity stretching and a passive positioning program.

| **QUESTION #1: Choose one of the above clinical scenarios. Write a focused clinical question for that scenario that will help you organize a search of the clinical literature.** | | | | | |
| --- | --- | --- | --- | --- | --- |
|  | Excellent | Strong | Limited | Minimal | Not evident |
| a: Population | 6: Multiple relevant descriptors; e.g., "work-related injury," "female" or "acute," or " or “low-back pain”; e.g., "boy with hemiparesis" specific age group, gender, diagnosis, motor presentation | 4: One appropriate descriptor as above examples; e.g. "women” or “worker” or “low-back pain”; e.g. "hemiparesis" "boy" "10 year old" "post-stroke" | 2: A single general descriptor unlikely to contribute to search; e.g. "patient" |  | 0: None of the above present |
| b: Intervention | 6: Includes specific intervention of interest; (intervention could be a diagnostic technique);   - manual therapy - specific individual components of manual therapy - combination of exercise and manual therapy; - task-specific strengthening |  | 2: Mentions intervention but unlikely to contribute to search; e.g. "methods" "options" "treatments" |  | 0: None of the above present |
| c: Comparison | 6: Identifies specific alternative of interest; e.g. “no manual therapy”; “low intensity stretching” |  | 2: Mentions comparison but unlikely to contribute to search; e.g. "alternate methods" |  | 0: None of the above present |
| d:  Outcome | 6: Outcome that is objective and meaningful to patient or patient case (if question is diagnostic, should relate to diagnosis trying to detect); e.g.return to work, pain reduction, injury prevention; e.g. selective motor control or functional use of paretic extremities, walking velocity | 4: Non-specific outcome:   - recovery - spasticity - tone - strength | 2: Reference to outcome, but so general as to be unlikely to contribute to search   - effects - change the outcome - effective - improvement - success - change the outcome |  | 0: None of the above present |

| **QUESTION #2: Where might you find answers to this and other similar clinical questions? Name as many possible sources of information as you can – not just the ones you think are ‘good’ sources. Describe the advantages and disadvantages of each type of information source you have listed.** | | | | | |
| --- | --- | --- | --- | --- | --- |
|  | Excellent | Strong | Limited | Min | Not evident |
| a:  Variety of Sources | 6: At least four types of sources listed. Types include:   - electronic databases of original literature [Medline(PubMed/Ovid), CINAHL] - discipline specific databases (Hooked on Evidence, PEDro) - journals (JAMA, NEJM, incl. access through library) - text book (Merck, Harrisons, monographs) - Systematic Reviews (Cochrane) - EBM publications or databases of pre-appraised information (Best Evidence, InfoRetriever, DynaMed, EBM, ACPJC, EBP, Clinical Evidence) - Medical website (MDConsult, PraxisMD, SumSearch) - General internet search (google, yahoo) - Clinical Guidelines (Guideline Clearinghouse, - Professional Organization (AAFP, La Leche League, NIH website) - People (colleague, consultant, attending, librarian) | 4: Three types of sources listed. | 2: Two types of sources listed. |  | 0: No variety. Only one source listed, or all sources of same type. |
| b: Convenience | 6: Discussion includes at least 2 specific issues related to convenience, or mentions the same issue while discussing two different sources. Issues may include:   - Cost (e.g. "free," "subscription only") - Speed (e.g. "fast," "takes time") - Ease of search (e.g. "must know how to narrow search," "easy to navigate") - Ease of use (e.g. "concise" and "NNTs already calculated") - Availability (e.g. "readily available online") | 4: Includes 1 specific issue/explanation related to convenience | 2: Mentions convenience involved in using one or more source, but without explanation  e.g. "convenient" or "easy" or "difficult" |  | 0: No mention of convenience |

#2 Continued

|  | Excellent | | | | Strong | Limited | | M | Not evident | |
| --- | --- | --- | --- | --- | --- | --- | --- | --- | --- | --- |
| c:  Clinical Relevance | 6: Discussion includes at least 2 specific issues related to relevance, or mentions the same issue while discussing two different sources. Issues may include:   - Clinically relevant outcomes - Written for clinical application (e.g. "pertinent" "info on adverse effects" or "has patient information sheets") - Appropriate specialty focus (e.g. "directed at PTs") - Information applicable to patient in question (e.g. "can go over details of this particular patient" or "most of studies are from Europe") - Includes specific interventions in question - Specificity (overview vs. targeted) (e.g. "can get basic information" or "more specialized") - Comprehensiveness of source (likelihood of finding an answer in that source) (e.g. "she can find anything" or "contains usable references" or "not likely to have answer to this question") | | | | 4: Includes 1 specific issue/explanation related to relevance | 2: Mentions relevance of using one or more source, but without explanation  e.g. "relevant" | |  | 0: No mention of relevance | |
| d:  Validity | 6: Discussion includes at least 2 specific issues related to validity, or mentions the same issue while discussing two different sources. Issues may include:   - Certainty of validity (e.g. quality is uncertain" or "has not been screened" or "needs to be critically appraised") - Evidence Based approach (e.g. "evidence based" or "Grade 1 Evidence" or "no references provided") - Expert bias (e.g. "usually just someone’s opinion") - Systematic approach - Peer review - Ability to verify - Standard of care (e.g. "accepted in medical community") - Enough information provided to critique validity (e.g. "abstract only" or "not available full-text") - Up-to-date/outdated (e.g. "most recent research") - Reliability – in the context of the degree of trust that can be places on the resource | | | | 4: Includes 1 specific issue/explanation related to validity | 2: Mentions validity of using one or more source, but without explanation  e.g. "good" "junk" | |  | 0: No mention of validity | |
| **QUESTION #3: What type of study (study design) would best answer your clinical question outlined in Q1 and why?** | | | | | | | | | | |
|  | | Excellent | Strong | Limited | | | Minimal | | | Not evident |
| a:  Study Design | | 12: Names one of the best sources:   - Randomized Controlled Trial - Randomized Trial - Systematic Review; - Meta-Analysis - Randomized, Double Blinded Clinical Trial | 9: Describes but does not call by name one of the best sources as above   - comparing two groups, one gets treatment, other gets placeo - double blind study | 6: Describes or names a less desirable study design:   - Cohort study - Prospective clinical trial - meta-analysis of such studies - Longitudinal or prospective | | | 3: Describes or names a poor study design to answer a treatment question:   - case control, cross sectional study, case report, "retrospective" - Or describes a study with insufficient detail to identify a design: e.g. a comparative study | | | 0: None of above present |
| b: Justification | | 12: Includes well-reasoned justification that reflects understanding of the importance of randomization and/or blinding. Explicitly connects randomization to reduction of confounding and/or blinding to observer or measurement bias.  e.g. "An RCT will attempt to avoid any bias which would influence the outcome of the study through randomization" OR "best suited for therapy questions because it reduces bias and controls for confounding factors." | 9: Justification is present, and touches on issues related to randomization and/or blinding, but less clearly articulated  e.g. "groups should be similar" or "try to eliminate confounding factors" or "avoid selection bias" or "to be objective" or "to eliminate bias" | 6: Justification is present, and raises legitimate issues unrelated to randomization or blinding, such as cost effectiveness, ethical concerns, recall bias.  May mention randomization or blinding but without explanation. (e.g. "best in a random and blind setting")  e.g. "chart reviews provide lots of data without much cost" | | | 3: Attempted justification, but arguments are non-specific and do not demonstrate understanding of the relationship between the design and various threats to validity  May mention randomization or blinding but without explanation. (e.g. "best in a random and blind setting")  e.g. "to ensure quality" or "to reduce potential conflicts" or "to compare" | | | 0: None of above present |

| **QUESTION #4: If you were to search Medline, CINAHL or any other database for original research to answer your clinical question related to the scenario you selected for Question 1, describe the search strategy you might use. Be as specific as you can about the search terms and search fields you would use. Explain your rationale for taking this approach. Describe how you might limit your search if necessary and explain your reasoning.** | | | | | |
| --- | --- | --- | --- | --- | --- |
|  | Excellent | Strong | Limited | M | Not evident |
| a:  Search Terms | 8: 3 or more terms that reflect patient, intervention, comparison, and outcome (PICO) being considered | 6: 2 terms from PICO | 3: 1 term from PICO |  | 0: Not present |
| b:  Tags/Strategy | 8: Description of search strategy reflects understanding that articles in database are indexed by more than one field.  Discusses one or more field/index/tag by name (MeSH, Title Word, Publication Title, language, Keyword, author, Journal title, use of boolean operators, etc.)  **AND** provides plausible rationale for search strategy using 1 or more of these indices  e.g. "keyword is less specific than MESH" | 6: Names 1 or more field or index category but does not provide plausible defense of search strategy based on this knowledge  e.g."I would do a keyword search…followed by…"  “I would use terms … in this way” | 3: weak description of strategy, no name given to tags, or overtly misguided strategy  e.g. “I would use terms ….” [no description of strategy] |  | 0: No evident understanding that articles "tagged" by different fields or indices |
| c:  Delimiters | 8: Describes more than one approach to limiting search (e.g., "limit to human" or "adult" or "English"), names a specific publication type, or describes of Clinical Queries in PubMed, or the use of Boolean operators or search combinations or includes terms related to an optimal study design (e.g. randomized) or suggests use of subheadings  * NOTE: If the subject includes the name of the index when describing a delimiter (e.g. "check language as English") then we give credit for a tag as well as a method of delimiting. | 6: Describes only 1 common method of limiting search  e.g. describes ways to narrow search using keywords but no other strategies listed | 3: provides weak explanation or description of use of limiters/narrowing search |  | 0: No valid techniques for limiting a search listed |

| **QUESTION #5: When you find a report of original research on this question or any others, what characteristics of the study will you consider to determine if it is relevant? Include examples. Questions 6 and 7 will ask you how to determine if the study is valid, and how important the findings are. For this question, please focus on how to determine if it is really relevant to your practice.** | | | | |
| --- | --- | --- | --- | --- |
|  | Excellent | Strong | Limited | Not evident |
| a:  The Question | 12: **Well-reasoned and thoughtful discussion of the relevance of the independent and dependent variables used in the study including examples/specific reasons.**  May discuss (well-reasoned and thoughtful):   - the feasibility of the test or intervention - "the test might work but if my practice can’t afford to buy the machine it doesn’t matter" - the patient or disease-oriented nature of the outcome - "did they measure children’s ability to use improved function in play activities?" - the congruence between the operational definition and the research question e.g. "whether their method of measuring the outcome is a realistic representation of the outcome we care about" | 9: **Less thoughtful discussion of the relevance of the independent and dependent variables used in the study. May include specific concepts or examples without clear rationale.**  May refer to same items listed in ‘excellent’ but without demonstrating depth of understanding. | 5: **Response implies consideration of how well the study addresses the question at hand, but offers little discussion about why this may be important**   - e.g. "what are the variables?"; - "does it answer my question?"; - "the outcome measure"; - "the purpose of the study"; - "will it impact my practice?"; - "length of follow-up" | 0: No discussion of the research question and variables used to answer it. |
| b: Description of Subjects | 12: **Includes both:**  A clear expression of the importance of the link between the study subjects and target population.  AND  At least one example of a relevant disease or demographic characteristic   - e.g. "were the patients similar to mine in terms of age and race?" or - "was it a hospital or clinic sample like my patients?" or - "did patients have same level of disease severity as my patient?" or - "did selection or inappropriate inclusion criteria result in a study population that differs from mine by race, age, etc" | 9: **Includes one but not both:**  A clear expression of the importance of the link between the study subjects and target population  **OR**  At least one example of a relevant disease or demographic characteristic  e.g. "is the patient like mine?" or "education level of population" | 5: **Response implies consideration of the study subjects, but offers no discussion of the connection between study subjects and target population or specific characteristics of the sample**   - e.g. "is it an appropriate sample?" or - "what was the response or participation rate?" or - "what were the exclusion criteria?" or - "selection bias" or - "setting" or - "where study was conducted" | 0: No discussion of the characteristics of the research subjects. |

NOTE: RESPONSES TO QUESTIONS 5, 6, AND 7, CAN BE APPLIED TO ANY PORTION OF THE GRADING RUBRIC FOR THOSE ITEMS

| **QUESTION #6: When you find a report of original research related to your clinical question or any others, what characteristics of the study will you consider to determine if its findings are valid? (You've already addressed relevance, and question 7 will ask how to determine the importance of the findings. For this question, please focus on the validity of the study.)** | | | | | |
| --- | --- | --- | --- | --- | --- |
|  | Excellent | Strong | Limited | Minimal | Not evident |
| a: Internal Validity | 24: Lists or describes at least 5 issues important to internal validity, such as:   - Appropriateness of study design - Adequacy of blinding - Allocation concealment - Randomization of group assignment - Invalid or biased measurement ("followed own protocol?") - Importance of comparison or control group - Intention to treat analysis - Consideration of appropriate covariates ("were other relevant factors considered?") - Conclusions consistent with evidence ("do the results make sense?") - Importance of follow-up of all study participants - Appropriate statistical analysis - Sample size / Power - Sponsorship - When study was conducted - Confirmation with other studies - Valid outcome measures | 18: Identifies 3-4 specific issues as above. | 10: Identifies 2 specific issues as above. | 5: Mentions internal validity or lists one specific concept from examples above. | 0: None of the above present |

**NOTE: RESPONSES TO QUESTIONS 5, 6, AND 7, CAN BE APPLIED TO ANY PORTION OF THE GRADING RUBRIC FOR THOSE ITEMS**

| **QUESTION #7: When you find a report of original research which relates to your clinical question or any others, what characteristics of the findings will you consider to determine their magnitude and significance (clinical and statistical)?** | | | | | |
| --- | --- | --- | --- | --- | --- |
|  | Excellent | Strong | Limited | Minimal | Not evident |
| a: Magnitude | 12: Response must clearly discuss both:   - clinical significance ("what is the clinical significance?" or "how large a difference was found?", does change exceed MCID)   AND   - example(s) of effect size measurements (e.g., specificity, sensitivity, likelihood ratio of a test, number needed to treat, relative risk, absolute risk reduction, mean difference for continuous outcomes, positive or negative predictive value) | 9: Response discusses one but not both:   - clinical significance ("what is the clinical significance?" or "how large a difference was found?")   OR   - example(s) of effect size measurements (e.g., specificity, sensitivity, likelihood ratio of a test, number needed to treat, relative risk, absolute risk reduction, mean difference for continuous outcomes, positive or negative predictive value) | 5: Response only suggests consideration of clinical significance or size of effect.   - e.g. "does it matter?" "will it impact my practice" or - e.g. mentions “Minimal Clinically Important Difference” but does not explain how this value would be used to determine clinical significance) |  | 0: None of the above present |
| b:  Statistical Significance | 12: Well-reasoned and thoughtful discussion of the indices of statistical significance, including at least 2 specific examples of important related concepts such as:   - p-values - confidence intervals - power - precision of estimates - Type 1 or Type 2 error | 9: Lists more than one concept (as above) with insufficient or absent discussion (e.g. "p-value and confidence intervals")  OR  Lists and discusses only one concept (e.g. "p-value less than <.05") | 5: Mentions need to assess statistical significance or names only one concept from above without further discussion (e.g. "p-values", “statistically significant”) |  | 0: None of the above present |

**NOTE: RESPONSES TO QUESTIONS 5, 6, AND 7, CAN BE APPLIED TO ANY PORTION OF THE GRADING RUBRIC FOR THOSE ITEMS**

| **QUESTION #8: For the clinical scenario that you chose, list up to two questions that you would ask the patient/family to gain a better understanding of his or her personal preferences and/or circumstances regarding your clinical question.** | | | | | |
| --- | --- | --- | --- | --- | --- |
|  | Excellent | Strong | Limited | Minimal | Not evident |
| a: Question 1 | 8: Question is likely to elicit important information about patient preferences, values, circumstances, expectations, and/or motivations that are will directly impact clinical care. |  | 4: Question is general but addresses issues relevant to understanding the patient’s perspective | 2: Question is general and does not address issues specific to the patient's perspectives  e.g. Standard question from subjective evaluation not specific to patient perspectives  e.g. Yes/No or factual questions that are unlikely to elicit details about patient perspective | 0: No question or not an actual question   - “past medical history” or - “preferences” |
| b: Question 2 | 8: same as above but elicits different information than the first question (otherwise 0) |  | 4: same as above but elicits different information than the first question (otherwise 0) | 2: same as above but elicits different information than the first question (otherwise 0) | 0: No question or not an actual question |

**Item #9 was dropped from the final version of the modified FT due to poor psychometric performance. See Part B of the Appendix for more information.**

| **QUESTION #10:**  **A study of the diagnostic accuracy of exercise treadmill testing (ETT) in diagnosing of coronary artery disease (CAD) included 96 women with suspected CAD, 29 of whom were subsequently determined to have CAD (>50% stenosis in one or more coronary vessels). Of those with CAD, 9 had an abnormal ETT. Of the 67 patients determined not to have CAD, 32 had an abnormal ETT.**  *** Alternative text using natural frequency values:**  A study of the diagnostic accuracy of exercise treadmill testing (ETT) in diagnosing of coronary artery disease (CAD) included 120 women with suspected CAD, 30 of whom were subsequently determined to have CAD (>50% stenosis in one or more coronary vessels). Of those with CAD, 10 had an abnormal ETT. Of the 90 patients determined not to have CAD, 30 had an abnormal ETT. | | | | | |
| --- | --- | --- | --- | --- | --- |
|  | Excellent | Strong | Limited | Minimal | Not evident |
| a:  Based on these results, the sensitivity of ETT for CAD is… | 4: 0.31; 31%; 9/29  *4: 0.33; 33%; 10/30 | 3: Within 5%: 26-36% |  |  | 0: No answer or wrong answer |
| b: Based on these results, the positive predictive value of ETT for CAD is… | 4: 0.22; 22%; 9/41  *4: 0.25; 25%; 10/40 | 3: Within 5%: 17-27% |  |  | 0: No answer or wrong answer |
| c: Based on these results, the likelihood ratio positive for an abnormal ETT for CAD is… | 4: 0.65; 31/47  *4: 1.0; 0.333/1-0.666 | 3: Within 5%: 0.60-0.70 |  |  | 0: No answer or wrong answer |

Rounding is acceptable (*e.g.* 21.9 to 22 is acceptable)

| **QUESTION #11: A recent randomized trial of pregnant women with incontinence found that after pelvic floor training 20% had incontinence compared to 32% in a control group at 3 mos after delivery. Alpha level for the study was set at the 0.05 significance level.**  *** Alternative text using natural frequency values:**  A recent randomized trial of pregnant women with incontinence found that after pelvic floor training 20% had incontinence compared to 30% in a control group at 3 mos after delivery. Alpha level for the study was set at the 0.05 significance level. | | | | | |
| --- | --- | --- | --- | --- | --- |
| a: The absolute risk reduction for recurrent events is… | 4: 0.12; 12%  *4: 0.10; 10% | 3: within 2%: 10-14% |  |  | 0: No answer or wrong answer |
| b: The relative risk reduction for recurrent events is… | 4: 0.38; 38%; 12/32  *4: 0.33; 33%; 10/30 | 3: within 2%: 36-40% |  |  | 0: No answer or wrong answer |
| c: The number needed to treat (NNT) to prevent one recurrent event is… | 4: 9; 1/0.12  *4: 10; 1/0.10 | 3: within 1: 8-10 |  |  | 0: No answer or wrong answer |
| d: The p-value indicating statistically significant difference between the groups would be… | 4: <0.05 | 3: 0.05 |  |  | 0: No answer or wrong answer |

| **QUESTION #12: The same study described in question 11 revealed a relative risk of incontinence of 0.61 for the women receiving pelvic floor training. This suggests that pelvic floor training treatment reduces risk for incontinence. We wonder if this difference is statistically significant, so we look at the confidence interval. Give an example of a confidence interval that would support the conclusion that the rate of incontinence was indeed (statistically) different for these two treatment groups.**  *** Alternative text using natural frequency values:**  The same study described in question 11 revealed a relative risk of incontinence of 0.66 for the women receiving pelvic floor training. This suggests that pelvic floor training treatment reduces risk for incontinence. We wonder if this difference is statistically significant, so we look at the confidence interval. Give an example of a confidence interval that would support the conclusion that the rate of incontinence was indeed (statistically) different for these two treatment groups. | | | | | | | |
| --- | --- | --- | --- | --- | --- | --- | --- |
|  | Excellent | Strong | Limited | | | Minimal | Not evident |
| a | 4: Indication that any CI that does not include 1 would indicate statistical significance |  | |  |  | | 0: Other |

| **QUESTION #13: Which study design is best for a study about diagnosis?** | | | | | |
| --- | --- | --- | --- | --- | --- |
| a | 4: Cohort Study; Cross Sectional Study; Comparison with gold standard; systematic review |  |  |  | 0: Other |

| **QUESTION #14: Which study design is best for a study about prognosis?** | | | | | |
| --- | --- | --- | --- | --- | --- |
| a | 4: Cohort; prospective; longitudinal; systematic review |  |  |  | 0: Other |

**Part B. Deleted item (#9)**

Original question and scoring rubric

| **QUESTION #9: For the clinical scenario that you chose, describe how clinical expertise might influence your ultimate plan for that patient.** | | | | | |
| --- | --- | --- | --- | --- | --- |
|  | Excellent | Strong | Limited | Minimal | Not evident |
| a: | 8: Describes an insightful opinion of how clinical expertise might influence the action plan for one of the scenarios. .  'I would consider my personal experience using manual therapy with persons with work', 'my own confidence with different manual skills may influence what I recommend', 'I would ask another clinical expert for their opinion' |  | 4: Comment is excessively general   - e.g. 'I would base my decision on what I had seen in the past’ or - Statement explicitly states that care would be determined based exclusively on clinical expertise |  | 0: No comment or demonstrates low appreciation of how to integrate clinical expertise and other sources of evidence 'I would do what I know works' OR  Discusses research evidence and not clinical expertise evidence |
